# The Effect of Collargolum on the Bacteria That Accompany the Tubercle Bacillus

**Published:** 1905-01

**Authors:** Max Behr

**Affiliations:** Assistant at Koehler’s Holsterhausen Sanatorium, Werden an der Ruhr, Germany


### ABSTRACTS.

### The Effect of Collargolum on the Bacteria That Accompany the Tubercle Bacillus.

MAX BEHR,
Assistant at Koehler's Holsterhausen Sanatorium, Werden an der Ruhr, Germany.

THE extensive literature on the use of collargolum in the most varied infectious processes incited Behr to try it in mixed phthisis infection. He used the rectal method lately recommended by Loebl and simultaneously administered it by the mouth. A tablespoonful of a 1 per cent. solution was exhibited by mouth twice daily in coffee, tea or cocoa; per rectum 1 oz. of 1 per cent. solution was given daily. Defecation and a cleansing irrigation preceded every injection. Thus every patient received about 7½ grains of the silver per day.

The treatment was given to those phthisis patients in whose sputum repeated careful examination had demonstrated an abundance of streptococci and staphylococci. Koch and his followers believe that mixed infection is present in the majority of phthisis patients and that the pus producing cocci are more injurious even than the tubercle bacilli, causing the tissue destruction in the lungs; while the Leyden school considers it unimportant. Behr attacks the question from the practical standpoint, observing what effect the treatment had on the cocci.

He records 14 cases, of which 11 had phthisis in the first and 3 in the second stage. Collargolum was given for an average of 110 days. The expectoration diminished or disappeared in 10

cases; the number of cocci was decreased in 8 cases. Three patients discontinued the treatment on account of anorexia and the like. The general results, especially as shown in the increase of weight, were very satisfactory.

Behr does not agree with Loebl that 14 days is the limit of time for the enemata. In fact, in cases in which there were by-effects at first, the remedy was well borne when persisted in.

In one case of uncertain diagnosis the sputum contained enormous masses of pus cocci, and Behr was astonished at the result of four weeks' collargolum treatment. The amount of cocci greatly diminished and the general condition markedly improved. He therefore also recommends a trial of the drug in non-tubercular suppurative pulmonary processes. His observations have not been extensive enough to enable him to affirm with certainty that it combats the tuberculosis itself; but it does influence the pus organisms that so often accompany the tubercle bacilli.

